# A Rare Presentation of Myocardial Bridging With Heart Failure Symptoms

**DOI:** 10.7759/cureus.56462

**Published:** 2024-03-19

**Authors:** Riya Mohan, Pradeep Dayanand, Sajid Mirza, Waqas Ghumman, Cristiano Faber

**Affiliations:** 1 Internal Medicine, University of Miami JFK Medical Center, Atlantis, USA; 2 Internal Medicine, University of Miami, JFK Regional Campus, Atlantis, USA; 3 Cardiology, University of Miami JFK Medical Center, Atlantis, USA; 4 Cardiothoracic Surgery, JFK Medical Center, Atlantis, USA

**Keywords:** myocardial bridge, congenital anomaly, angina, heart failure, ventricular dysfunction, coronary artery bypass graft

## Abstract

Myocardial bridging is an under-recognized cause of angina. This congenital anomaly occurs when a segment of the epicardial coronary artery has a short intra-myocardial course. A significant intra-myocardial course may lead to ischemia, causing anginal symptoms. In this case report, we discuss a rare presentation of myocardial bridging with symptoms of heart failure. The pathology led to a marked degree of ventricular dysfunction and a significant drop in cardiac output (CO), and the patient had severe exertional dyspnea and functional limitations. The ischemic workup with diagnostic imaging and angiograms failed to explain the severity of symptoms, which were only evident in hemodynamic studies and cardiopulmonary exercise testing.

## Introduction

Myocardial bridging occurs when an epicardial coronary vessel has an unusual intra-myocardial course [1]. Compression of the vessel segment during systole results in decreased myocardial perfusion and transient ischemia. Myocardial bridging had initially been thought to be a benign, incidental finding. However, the presence of this anomaly has increasingly been recognized as a cause of angina, myocardial infarction (MI), and heart failure [2,3].

## Case presentation

A 76-year-old man presented to the emergency room with a history of progressive exertional chest discomfort and dyspnea for three months. He had an acute worsening of symptoms in the week prior, which prompted him to come to the emergency department. His past medical history was relevant for hypertension, paroxysmal atrial fibrillation, hyperlipidemia, and ischemic stroke. He was notably an active and functional individual prior to the onset of symptoms. The cardiovascular exam was unremarkable; he was clinically euvolemic without jugular venous distention or peripheral edema. Auscultation revealed a regular rate and rhythm with normal first and second heart sounds.

Investigations

An electrocardiogram (ECG) on admission showed normal sinus rhythm and a right bundle branch block. The echocardiogram showed no regional wall motion abnormalities, with normal systolic function and an ejection fraction (EF) of 55%. Grade I diastolic dysfunction was present. An exercise stress test was then performed, which resulted in abnormal results as his exercise capacity was 1 metabolic equivalent (1 MET), with the patient developing shortness of breath and severe fatigue after two minutes of the Bruce protocol. The study was then converted to a pharmacologic stress test with Regadenoson. A myocardial single photon emission computed tomography (SPECT) study revealed a medium-sized, anterolateral partially reversible myocardial perfusion defect. Coronary angiography was subsequently performed; the findings were notable for borderline stenosis of an obtuse marginal branch of the circumflex artery with a fractional flow reserve (FFR) of 0.96. The only other significant finding was the myocardial bridging of the mid-left anterior descending (LAD) artery.

Despite the benign findings from his cardiac catheterization and subsequent outpatient workup, the patient returned to the outpatient clinic due to worsening symptoms upon exertion. We performed a cardiopulmonary stress test to further evaluate the etiology of his dyspnea and functional capacity, as the ischemic workup and imaging studies, including angiography, did not demonstrate significant functional limitations. The patient exercised for four minutes on a cycle ergometer with a peak work rate of 29 watts. His respiratory exchange ratio was acceptable at 1.16. Maximum oxygen uptake (VO_2_ max) adjusted for weight was markedly reduced to 9.5 ml/kg/min (52% of predicted). His pulse oxygen was 93% of what was predicted, though the patient demonstrated chronotropic incompetence by achieving a peak heart rate of only 84 bpm (52% predicted). Anaerobic threshold (AT) occurred at 9.4 ml/kg/min, 51% of the predicted VO_2_ max. His breathing reserve was normal; however, his peak ventilation/carbon dioxide production (VE/VCO_2_) was markedly elevated at 51.

Right heart catheterization (RHC) with exercise and fluid challenge was then performed to further explore the patient’s cardiac limitations. It revealed a marked limitation in chronotropic reserve and a transient, severe drop in cardiac output (CO) with exercise, as outlined below (Table 1). It is pertinent to note that the cardiac index (CI) significantly dropped from 2.59 L/min/m^2^ at rest to 1.37 L/min/m^2^ at peak exercise after two minutes, meeting the criteria for cardiogenic shock; also, the CO and CI made a remarkable recovery to within normal levels after five minutes of rest, proving the transient nature of the causative lesion.

**Table 1:** Right heart catheterization parameters Bpm: beats per minute; RA: right atrium; RV: right ventricle; PA: pulmonary artery; PCWP: pulmonary capillary wedge pressure; NIBP: non-invasive blood pressure; SaO2: oxygen saturation on blood analysis; AO: aortic; FCO: fick cardiac output; CI: cardiac index; SVR: systemic vascular resistance; PVR: pulmonary vascular resistance; WU: woods unit; L: liter

At rest	At peak exercise after 2 minutes	After 5 minutes of rest	After 1 L Fluid bolus
Heart rate: 64 bpm	Heart rate: 78 bpm	Heart rate: 66 bpm	Heart rate: 68 bpm
Pressures: systolic/diastolic/mean (mmHg) RA 5/4/2 RV 30/6 PA 32/13/19 PCWP 5/6/5 NIBP 156/90/111	Pressures: systolic/diastolic/mean (mmHg) PA 39/13/22 PCWP 6/7/6 NIBP 150/88/104	Pressures: systolic/diastolic/mean (mmHg) PA 26/8/14 PCWP 5/6/5 NIBP 156/86/108	Pressures: systolic/diastolic/mean (mmHg) PA 31/11/18 PCWP 9/13/7 NIBP 154/84/106
SaO_2_ (%): RA 74.9 PA 73.6 AO 96.0	SaO_2_ (%): PA 53.5 AO 96.0	SaO_2_ (%): PA 74.5 AO 95.0	SaO_2_ (%): PA 75.7 AO 97.7
FCO/CI: (L/min)/(L/min/m^2^) 6.02/2.59 SVR 1448 dynes PVR 2.32 WU	FCO/CI: (L/min)/(L/min/m^2^) 3.17/1.37 PVR: 5.04 WU	FCO/CI: (L/min)/(L/min/m^2^) 6.58/2.83 PVR 1.37 WU	FCO/CI: (L/min)/(L/min/m^2^) 6.14/2.65 PVR 1.79 WU

In summary, it was noted that there was only a minimal change in left and right filling pressures during exercise but a marked drop in Fick CO and CI. The transient symptoms were attributed to transient myocardial ischemia, due to known myocardial bridging.

In our patient, the myocardial bridging caused significant dyspnea on exertion (NYHA III Functional Class), leading to multiple physician visits and recurrent hospitalizations.

Treatment

It was concluded that the patient was symptomatic despite maximally tolerating optimum medical therapy with beta-blockade and calcium channel blockers (diltiazem). A shared decision with the patient was made to pursue single-vessel coronary artery bypass grafting (CABG), left internal mammary artery (LIMA), and LAD. After the surgery, the patient improved symptomatically and was discharged home after a short and uncomplicated hospital course. He underwent cardiac rehabilitation with a return of exercise tolerance and functional capacity to baseline prior to the onset of symptoms.

## Discussion

The myocardial bridge is usually an incidental finding noted on cardiac catheterization or during the autopsy; the overall prevalence rates were reported in multiple studies from 5% to 86%, with a mean of 25% [2]. Most bridges were located on the LAD [1]. This used to be considered a benign finding, and most patients are asymptomatic; however, several recent studies have reported its association with a wide variety of conditions, such as acute coronary syndromes, coronary spasms, ventricular septal ruptures, arrhythmias, exercise-induced atrioventricular conduction blocks, transient ventricular dysfunction, and sudden cardiac death [3-6].

The degree of myocardial ischemia appears out of proportion to the degree of compromise in coronary blood flow by the bridging. Because the majority of the coronary filling should occur in diastole, systolic compression of the bridge segment should only have a minimal effect on the total effective myocardial perfusion [6]. However, factors such as tachycardia, decreased systolic blood pressure, and coronary vasospasm may exacerbate the hemodynamic significance of this phenomenon [7]. Our patient presented with marked cardiac limitation on minimal exercise but was asymptomatic on rest. He had an inconclusive ischemic workup with imaging studies and coronary angiography, which failed to reveal the hemodynamic changes with exercise and the functional significance of the bridge segment; this was evident only on RHC with exercise and cardiopulmonary exercise testing.

Medications remain the first-line therapy. Beta-blockers and non-dihydropyridine calcium channel blockers are preferred due to their negative ionotropic and chronotropic effects [2]. Nitrates are contraindicated in patients with myocardial bridging [8]. Surgical options include surgical myotomies and CABG [2]. Percutaneous coronary intervention (PCI) has been done for symptom management refractory to medical therapy [9]. However, multiple cases of coronary perforation [10] and stent fracture [2] have been reported, and the role of PCI remains controversial. Our patient had significant symptomatic improvement post-CABG with a return of exercise tolerance and functional capacity.

The present case demonstrates the importance of recognizing the myocardial bridge as a potential cause of cardiac symptoms. In our patient, it caused significant cardiac limitation, which was evident in hemodynamic studies with RHC and cardiopulmonary exercise testing. Cardiologists should be vigilant about this condition as a cause of ischemic symptoms, especially if no fixed obstructive stenosis is identified. If the patient remains symptomatic with maximal medical therapy, CABG may be considered.

## Conclusions

The myocardial bridge was thought to be an incidental finding. However, this can lead to angina, arrhythmias, and symptoms of heart failure and should be recognized as a potential cause of cardiac limitation. Physicians should be vigilant about this condition as a cause of ischemic symptoms, especially if no fixed obstructive stenosis is identified in imaging studies. Treatment options include optimal medical management and surgical options. CABG may be considered if the patient remains symptomatic with maximal medical management.
